# Enzyme-Digested Edible Bird’s Nest (EBND) Prevents UV and Arid Environment-Induced Cellular Oxidative Stress, Cell Death and DNA Damage in Human Skin Keratinocytes and Three-Dimensional Epithelium Equivalents

**DOI:** 10.3390/antiox12030609

**Published:** 2023-03-01

**Authors:** Dongliang Wang, Naohiro Shimamura, Mai Mochizuki, Taka Nakahara, Katsuhisa Sunada, Li Xiao

**Affiliations:** 1 Beijing Xiaoxiandun Biotechnology Co., Ltd., No. 150, Guanzhuang Road, Changying Town, Chaoyang District, Beijing 100020, China; 2 Hebei Edible Bird’s Nest Fresh Stew Technology Innovation Center, Bazhou Economic Development Zone, Langfang 065700, China; 3 Department of Dental Anesthesiology, School of Life Dentistry at Tokyo, The Nippon Dental University, 1-9-20 Fujimi, Chiyoda-ku, Tokyo 102-8159, Japan; 4 Department of Life Science Dentistry, The Nippon Dental University, 1-9-20 Fujimi, Chiyoda-ku, Tokyo 102-8159, Japan; 5 Department of Developmental and Regenerative Dentistry, School of Life Dentistry at Tokyo, The Nippon Dental University, 1-9-20 Fujimi, Chiyoda-ku, Tokyo 102-8159, Japan; 6 Department of Pharmacology, School of Life Dentistry at Tokyo, The Nippon Dental University, 1-9-20 Fujimi, Chiyoda-ku, Tokyo 102-8159, Japan

**Keywords:** edible bird’s nest, UVA, UVB, DSBs, 3D epithelium equivalents, keratinocytes, arid environment, γ-H2A.X, inflammation, sialic acid

## Abstract

The aim of this study is to investigate the repressive effects of enzyme-digested edible bird’s nest (EBND) on the combination of arid environment and UV-induced intracellular oxidative stress, cell death, DNA double-strand breaks (DSBs) and inflammatory responses in human HaCaT keratinocytes and three-dimensional (3D) epithelium equivalents. An oxygen radical antioxidant capacity assay showed that EBND exhibited excellent peroxyl radical scavenging activity and significantly increased cellular antioxidant capacity in HaCaT cells. When EBND was administered to HaCaT cells and 3D epitheliums, it exhibited significant preventive effects on air-drying and UVA (Dry-UVA)-induced cell death and apoptosis. Dry-UVA markedly induced intracellular reactive oxygen species (ROS) generation in HaCaT cells and 3D epitheliums as quantified by CellROX^®^ Green/Orange reagents. Once HaCaT cells and 3D epitheliums were pretreated with EBND, Dry-UVA-induced intracellular ROS were significantly reduced. The results from anti-γ-H2A.X antibody-based immunostaining showed that EBND significantly inhibited Dry-UVA-induced DSBs in HaCaT keratinocytes. Compared with sialic acid, EBND showed significantly better protection for both keratinocytes and 3D epitheliums against Dry-UVA-induced injuries. ELISA showed that EBND significantly suppressed UVB-induced IL-6 and TNF-α secretion. In conclusion, EBND could decrease arid environments and UV-induced harmful effects and inflammatory responses in human keratinocytes and 3D epithelium equivalents partially through its antioxidant capacity.

## 1. Introduction

Solar ultraviolet A (UVA) (320–400 nm) and UVB (280–320 nm) can reach the Earth and accelerate skin aging by injuring skin cells. As the main spectral component of solar UV radiation, UVA is known to trigger the production of reactive oxygen species (ROS) in skin cells [1,2,3]. ROS causes DNA damage such as DNA double-strand break (DSB) and injures intracellular membranes resulting in cell dysfunction and cell death in the skin [4,5]. Although among the solar UV, the amount of UVB is only 1/20 of UVA, it is about 600–1000 times more harmful than UVA. UVB triggers inflammation, gene mutation and immunosuppression in the skin through ROS products as well as UVA [6,7,8]. The above-mentioned harmful effects of UV can be worsened under an arid environment.

Skin keratinocytes are the first barrier against UV radiation, water loss and other environmental toxins. They are also the first target for the lesions caused by these environmental toxins. In our previous study, we demonstrated that arid environment-induced dehydration could cause oxidative stress, injure cellular membranes and induce apoptotic cell death in human keratinocytes [9].

An edible bird’s nest (EBN) is made from the solidified saliva produced by swiftlets. Biological studies have demonstrated that EBN extracts have high medicinal benefits which include anti-inflammatory and anti-aging effects [10,11,12]. EBN stimulates epidermal growth and cell proliferation [13] and moisturizes the skin [14]. However, little is known about the effect of EBN on UV and arid environment-induced lesions in skin keratinocytes. In the present study, we investigated the protective effects of EBN extracts on air-drying and UV-induced cell death, membrane injury, DNA damage, ROS production and inflammatory cytokines secretion in human keratinocytes and three-dimensional (3D) epithelium equivalents. Since sialic acid (SA) is the best-known bioactive compound in EBN, we used SA as the control reagent.

## 2. Materials and Methods

### 2.1. Preparation of Edible Bird’s Nest and SA

**Figure 1:**
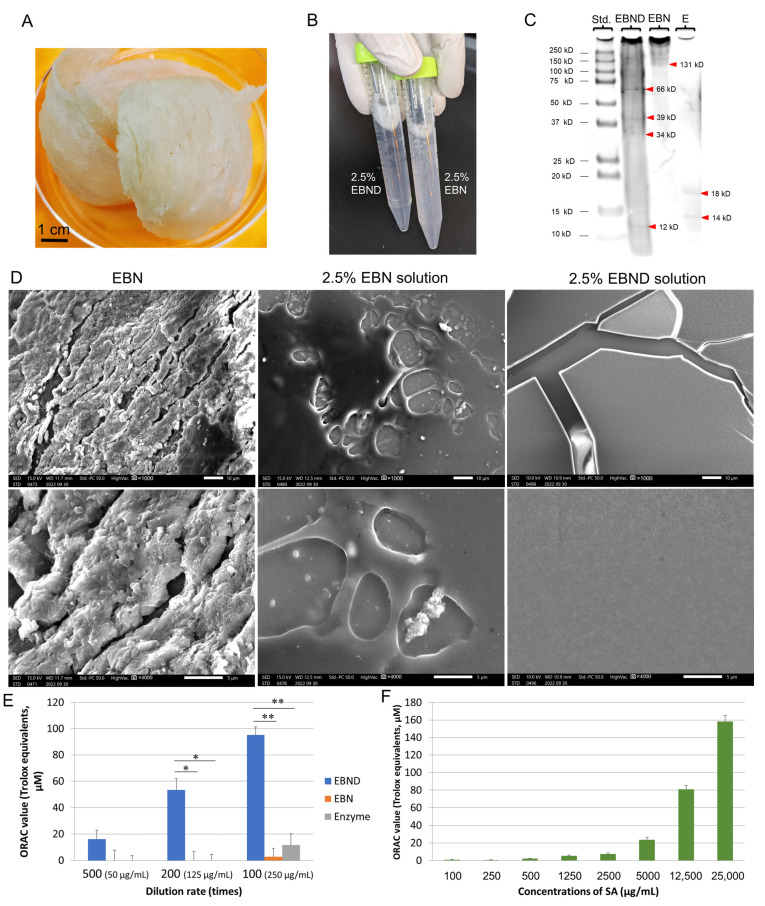
Protein distribution and peroxyl radical (ROO) scavenging activity of EBND: (**A**) Cup-shaped EBN (about 36 g in total); (**B**) Clarity of 2.5% EBND and 2.5% EBN; (**C**) Protein distribution and molecular weight of 2.5% EBND and EBN. Std.: protein standards; E: enzyme-only solution. Red triangles indicate bands and their molecular weight; (**D**) SEM images of EBN and 2.5% EBN and EBND solutions. Scale bars in the upper and lower panels indicate 10 and 5 µm, respectively; (**E**,**F**) Peroxyl radical scavenging activity (ORAC capacity) of EBND, EBN and SA. 2.5% (25 mg/mL) EBN, EBND and enzyme-only solutions were 1: 500 (50 µg/mL EBND or EBN, EBND 500), 1: 200 (125 µg/mL EBND or EBN, EBND 200) and 1: 100 (250 µg/mL EBND or EBN, EBND 100 or EBN 100) diluted by DDW. Their ORAC activity was measured by ORAC assay as described in Materials and Methods. Experiments were repeated 3–5 times independently. * *p* < 0.05, ** *p* < 0.01.

Imperial edible bird’s nest (EBN) (Figure 1A) (produced in Indonesia) was purchased from Kyouyou kokusai syouzi, Co. Ltd. (Chiba, Japan). EBN was cut into small pieces (about 2 × 2 cm^2^) and soaked in double distilled water (DDW) (25 mg/mL, 2.5% *w/v*) overnight. The next day, the soaked EBN was heated in a water bath at 95 °C for 20 min. After being cooled down to room temperature, the heated EBN mixture was pureed by a blender. Next, we digested the EBN solution by using a series of digestive enzyme mixtures (including gastro-intestinal enzymes, inorganic and organic compounds and others) according to our previous report [15,16]. The EBN solution first reacted with simulated gastric juice [15] at a volumetric rate of 125 (EBN solution): 1 (gastric juice) for 1 h at 37 °C on a bio-shaker. Then the solution further reacted with simulated bile (rate 112:1) and duodenal juice (rate 37:1) at 37 °C for another 1 h on a bio-shaker. At the end of the reaction, the enzyme-digested EBN solution (EBN digest, EBND) was centrifuged at 4 °C with a speed of 10,000 rpm for 15 min. The supernatant was filtered through 0.22-μm filters and kept at −80 °C until used. For the treatments of HaCaT cells, EBND solution was diluted to certain concentrations and added to the culture medium.

SA (015-26171, FUJIFILM Wako Pure Chemicals CO., Osaka, Japan) was dissolved in DDW at its saturated concentration, 50 mg/mL. The solution was filtered through 0.22-μm filters and kept at −20 °C until used.

### 2.2. SDS-PAGE Analysis

To determine protein distribution and the molecular weight of proteins in EBN and EBND, SDS-polyacrylamide gel electrophoresis (SDS-PAGE) was used to analyze the proteins in 2.5% (*w/v*) EBN and EBND. Briefly, 5 µL of EBN, EBND, digestive enzyme-only solution and protein standards (Precision Plus Protein™ WesternC™ Blotting Standards, 1610376, Bio-Rad, Hercules, CA, USA) were loaded into individual wells in a 12.5% polyacrylamide gel and ran under the constant current setting of 21 mA for 30 min with an electrophoresis apparatus (WSE-1010 cPAGE Ace, ATTO CO., Tokyo Japan). After electrophoresis, the gel was rinsed by DDW and stained with a quick CBB solution (299-50101, FUJIFILM Wako, Osaka, Japan) for 30 min. Clear bands were obtained by de-staining the gel with DDW. The protein gel was photographed with the iBright FL1500 Imaging System (Thermo Fisher Scientific, Tokyo, Japan). The protein bands were analyzed by iBright Analysis software (Thermo Fisher Scientific, Tokyo, Japan).

### 2.3. Scanning Electron Microscope (SEM) Analysis

One piece of dried half-cup EBN material was mounted on a stub and then coated with a thin layer of platinum in an ion sputter (E-1030, HITACHI, Tokyo, Japan). For the 2.5% EBN and EBND solutions, 1.5 µL solutions were dropped on cover glasses and air-dried overnight. The cover glasses were then mounted on the stub followed by ion coating the same as the dried half-cup EBN.

HaCaT cells (8 × 10^4^ cells/mL) were seeded into 35 mm dishes with a cover glass and cultivated for 24 h. Cells were then cultivated with EBND at 125 µg/mL for 2 h. After the medium was removed, cells were air-dried and irradiated with UVA for 5 min (10 J/cm^2^), and then re-soaked in the medium with EBND. Cells were further cultivated for 48 h. At the end of cultivation, HaCaT cells were pre-fixed for 30 min in 2.5% glutaraldehyde solution in PBS (-). After being washed with PBS (-), specimens were further fixed with 1% osmium tetroxide (OsO4) for 1 h. Samples were then dehydrated with ascending grades of ethanol. The specimens were substituted by t-butylalcohol and freeze-dried with a vacuum evaporator. The dried samples were mounted on stubs followed by ion coating. The preparations were examined at 10–15 kV using an SEM (JSM-IT200 InTouchScope™, JEOL Ltd., Tokyo, Japan) [9].

### 2.4. Oxygen Radical Absorbance Capacity (ORAC) Assay

An ORAC assay was performed according to our previous report [17]. Briefly, diluted EBN, EBND and digestive enzyme-only solutions at graded concentrations (25 µL) and standards (25 µL) were added into the wells of a microplate (black 96-well plates, Nunc^TM^ black microwell, Japan). The 2,20-Azobis(2-amidinopropane) dihydrochloride (AAPH) solution (25 µL; 12 mM final concentration) and fluorescein (150 µL; 70 nM final concentration) solution were mixed immediately before the reaction and added into the sample wells, rapidly using a multichannel pipette. The plate was immediately placed in a fluorescence microplate reader (SH-9000Lab, HITACHI, Tokyo, Japan), and the fluorescence intensities (Ex 480 nm, Em 520 nm) were recorded every 5 min for 90 min at 37 °C. The standard antioxidant, trolox (0, 12.5, 25, 50, 100, 200 µM) was also carried out in the same run.

For the measurement of cellular ORAC capacity, HaCaT cells (1 × 10⁶) were washed two times by PBS (-) and then lysed by using an EzRIPA Lysis kit (WSE-7420, ATTO CO., Tokyo, Japan). Protein concentrations of the cell lysate were measured with a protein quantification kit (Protein Quantification Kit-Rapid, Dojindo Molecular Technologies, Inc., Kumamoto, Japan). A total of 20 μL of each cell lysate with the same concentration of protein was mixed with 80 μL of 5% metaphosphoric acid. After being centrifugated (8000 rpm, 10 min), the supernatants were diluted by DDW at a rate of 1:10 and then used as the testing samples for ORAC assay.

### 2.5. Cell Culture

Immortalized human skin epidermal keratinocytes HaCaT were kindly provided by Professor Norbert E. Fusenig of Deutsches Krebsforschungszentrum (Heidelberg, Germany) [18]. Cells were maintained in Dulbecco’s modified Eagle’s medium (DMEM) (Gibco) supplemented with 10% FBS, 1% Gibco^®^ GlutaMAX™ Supplement (Thermo Fisher Scientific, Tokyo, Japan), in a 5% CO_2_-atmosphere at 37 °C.

### 2.6. 3D-Cultured Epithelium Equivalents

Three-dimensional epithelium equivalents were prepared with a Cellmatrix type I-A culture kit (Nitta Gelatin, Osaka, Japan) in a manner similar to our previous study [17]. In brief, type I-A collagen gel was mixed with 10% 10× concentrated MEM-alpha and 10% reconstruction buffer (2.2 g of NaHCO_3_ and 4.47 g HEPES in 100 mL 0.05 N NaOH). The gel mixture was added on top of an atelocollagen sheet (Integrin^®^ sheet, KOKEN Co., Ltd., Tokyo, Japan) which was placed in a 24-well culture plate (Corning Inc., Tokyo, Japan). After 10 min incubation at 37 °C, HaCaT cells (1 × 10^6^ cells/mL) were seeded on the collagen gel substrate and the medium was changed to KSR medium (MEM-alpha containing 15% KnockOut™ serum replacement and 1% Gibco^®^ GlutaMAX™ Supplement) containing 5% FBS. One day later, the medium was replaced with KSR medium containing 1% FBS. After further cultivation for 1–4 days, the culture surfaces were exposed to air in the incubator. The culture medium was replaced with KSR medium without FBS and incubated for another 2–4 weeks.

### 2.7. Air-Drying and UV Irradiation

HaCaT cells were first cultivated with EBND or SA at different concentrations for 2 h. Then, the culture medium was completely sucked up by an air pump (AP-115N, Iwaki, Tokyo, Japan) of speed as high as 13–15 L/min for 5 s. Cells were left at room temperature (22–24 °C) under 35–40% moisture and irradiated by a LED UVA lamp (HLV2-24UV2-365, CCS, Co., Osaka, Japan) for 5 min or a UVB lamp (UVP LLC, Upland, CA, USA) for 10 s. The UVA dose was about 10 J/cm^2^. The UVB dose was about 16 mJ/cm^2^. Culture media with EBND or SA at the same concentrations were added to the cell monolayer after drying and UV irradiation.

The 3D epithelium equivalents were treated with EBND (125 mg/mL) or SA (1.25 mg/mL) for 48 h. Then, the culture medium was completely sucked up in the same way as the description above. The epitheliums were irradiated with UVA for 20 min at a dose of 40 J/cm^2^. Culture media with EBND or SA at the same concentrations were added to the epitheliums after air-drying and UV irradiation. The UV intensity was measured by a UV light meter (YK-35UV ST, SATOTECH, Co., Kawasaki, Japan).

### 2.8. Cell Viability Assay

HaCaT cells were seeded into 35 mm^2^ 4-chamber glass bottom dishes (Cellvis, CA, USA) or 24-well plates and cultivated for 24 h. Cells were then cultivated with EBND or SA at different concentrations for 2 h. After the medium was removed, cells were air-dried and irradiated with UVA for 5 min (10 J/cm^2^) or UVB for 10 s (16 mJ/cm^2^), and then re-soaked in the medium with EBND or SA. Cells were further cultivated for 48 h. At the end of cultivation, HaCaT cells were incubated for 3 h at 37 °C with fresh culture media supplemented with 10% PrestoBlue^®^ (*v*/*v*; A13261, Thermo Fisher Scientific, Tokyo, Japan). The fluorescence intensity of PrestoBlue^®^ was measured by using a microplate reader (SH-9000Lab, HITACHI, Tokyo, Japan) with excitation/emission at 560 nm/590 nm [17].

### 2.9. Apoptosis Detection Methods

#### 2.9.1. Annexin V/PI Staining

HaCaT cells (2 × 10^4^ cells/well) were seeded into 35 mm^2^ 4-chamber glass bottom dishes (627870, CELLVIEW CELL CULTURE DISH, Greiner Bio-One Co., Ltd., Tokyo, Japan) and cultivated for 24 h. Cells were then followed by air-drying and UVA irradiation in the same way as the description above. At the end of cultivation, cells were rinsed twice with PBS (-) and stained with a Tali™ Apoptosis Kit (A10788, Thermo Fisher Scientific, Tokyo, Japan) according to the manufacturer’s instructions. Apoptotic cells were observed by EVOS^®^ FL Cell Imaging System (Thermo Fisher Scientific, Tokyo, Japan). For quantitative analysis, cells were dissociated from the vessel by trypsinization and then stained by annexin V and PI provided by the Tali^TM^ Apoptosis kit. After staining, cells were analyzed by Tali™ Image-Based Cytometer (Thermo Fisher Scientific, Tokyo, Japan).

#### 2.9.2. Terminal Deoxynucleotidyl Transferase Deoxyuridine Triphosphate Nick End Labeling (TUNEL) Assay

Apoptotic cells in the epithelium were detected by ApopTag^®^ Plus Peroxidase In Situ Apoptosis Kit (S7101, Chemicon^®^, Sigma-Aldrich, Tokyo, Japan) according to the manufacturer’s protocol. Briefly, the 3D epithelium equivalents were fixed by 10% formalin neutral buffer solution (062-01661, FUJIFILM Wako Chemicals, Osaka, Japan) at 4 °C overnight. The samples were then dehydrated by a series of ethanol and xylene followed by paraffin embedding. The paraffin tissue blocks were sectioned into 10 μm-thick slices by a microtome. After deparaffinization and hydration, apoptotic cells in the sample slices were detected by reagents of the TUNEL assay provided by the in situ apoptosis kit.

### 2.10. Enzyme-Linked Immuno-Sorbent Assay (ELISA)

To determine the concentration of human IL-6 and TNF-α derived from HaCaT cells, culture supernatants were analyzed using commercially available ELISA Kits for human IL-6 (KE00139, Proteintech Group, Inc. Rosemont, USA) and TNF-α (KE00154, Proteintech). HaCaT cells (4 × 10^4^ cells/well) were seeded into 24-well plates and cultivated for 24 h in the maintenance medium. Cells were then treated with EBND and followed with air-drying and UVB irradiation the same as the description in the cell viability assay. At the end of cultivation, cell culture supernatants were collected and reacted with the first and secondary antibodies, streptavidin-HRP and detection solution according to the manufacturer’s instruction. The reaction was stopped using the stop solution (from the ELISA kits) and absorbance was read at 450 nm using a microplate reader (SH-9000Lab, Hitachi, Tokyo) [19].

### 2.11. Cellular ROS Detection

HaCaT cells (2 × 10^4^ cells/well) were seeded into a 35 mm imaging dish with four compartments (ibidi µ-Dish, Nippon Genetics Co., Ltd., Tokyo, Japan) and cultivated for 24 h. Cells were treated with EBND (125 µg/mL) for 2 h and then followed with air-drying and UVA irradiation (10 J/cm^2^) for 5 min. Immediately after UVA irradiation, cellular ROS generation in HaCaT cells was detected with the CellROX^®^ Orange and Green Reagent (Thermo Fisher Scientific, Tokyo, Japan) according to the manufacturer’s recommended protocol. ROS production in cells was observed by EVOS^®^ FL Cell Imaging System (Thermo Fisher Scientific, Tokyo, Japan). For quantitative analysis, cells were dissociated from the vessel by trypsinization and then stained by CellROX^®^ reagents. After staining, cells were analyzed by Tali™ Image-Based Cytometer (Thermo Fisher Scientific, Tokyo, Japan).

ROS production in 3D epithelium equivalents was also detected with the CellROX^®^ reagents. After final treatment, the epitheliums were stained with CellROX^®^ orange, green reagents and NucBlue™ Live ReadyProbes™ Reagent (Hoechst 33342) (R37605, Thermo Fisher Scientific, Tokyo, Japan) working solution in PBS (-) for 30 min. The samples were then fixed with 10% formalin neutral buffer solution overnight at 4 °C and followed by OCT-compound embedding and cryosection. Red and green fluorescence of the samples were observed with the EVOS^®^ FL Cell Imaging System. The mean signal intensities of red and green fluorescence were analyzed with Image J software according to previous reports [20,21].

### 2.12. Determination of γ-H2A.X Foci Formation

HaCaT cells (2 × 10^4^ cells/well) were seeded into a 35 mm imaging dish with four compartments (ibidi µ-Dish) and cultivated for 24 h. Cells were then followed with EBND treatment, air-drying and UVA irradiation. At 2 h after UVA irradiation, HaCaT cells were fixed in ice-cold 100% methanol. Cells were then incubated with serum blocking solution for 10 min to suppress the non-specific binding of IgG, and then incubated with saturating levels of anti-γ H2A.X (phospho S139) antibody (ab26350, Abcam, Tokyo, Japan) for one day at 4 °C. After carefully washing with 1% Triton X-100 in PBS (-), specimens were reacted with fluorochrome-conjugated secondary antibody (A11001, Thermo Fisher Scientific, Tokyo, Japan) diluted to 2 µg/mL in 1% Triton in PBS (-) with 1.5% normal blocking serum for 1 h in dark. The nuclei were stained with propidium iodide (PI). Samples were imaged and analyzed with a confocal laser scanning microscopy (LSM700, Carl Zeiss Microscopy Co., Ltd., Tokyo, Japan). For quantitative analysis, cells were dissociated from the vessel by trypsinization and then followed by anti--γ H2A.X antibody-based immunostaining. After staining, cells were analyzed by Tali™ Image-Based Cytometer (Thermo Fisher Scientific, Tokyo, Japan).

### 2.13. Statistical Analysis

Statistical analysis was carried out similarly to our previous report [22]. All data, expressed as the mean ± SD, were analyzed statistically by GNU PSPP Statistical Analysis Software (version 0.8.2-gad9374) (https://www.gnu.org/software/pspp/ (accessed on 24 January 2023)) and EZAnalyze Excel-based tools (http://www.ezanalyze.com/ (accessed on 24 January 2023)). One-way ANOVA analysis of the variance was followed by the Post Hoc tests (including Tukey’s test and Bonferroni Correction). Statistical significance was considered when *p* < 0.05. All experiments were repeated 3–5 times independently.

## 3. Results

### 3.1. Features of EBN Material and Solutions of EBN and EBND

Before starting a series of experiments, we first analyzed features of EBN material, 2.5% (*w/v*) EBN and EBND solutions by using SEM and SDS-PAGE methods. As shown in Figure 1A, EBN material exhibited a half-cup or shallow-cup-like shape with a light yellow-white color. Figure 1B showed that without enzymatic digestion, the 2.5% EBN solution exhibited muddiness. After being digested by a series of digestive enzyme mixtures, the 2.5% EBND solution showed much higher clarity. SEM analysis showed that the surface ultrastructure of EBN presented a congealed glue-like feature (Figure 1D, left panel), and when the 2.5% EBN solution was dried, it exhibited a smooth surface with some granules (Figure 1D, middle panel) suggesting that there are some impurities inside the solution. On the contrary, the dried 2.5% EBND solution formed a thin film-like membrane with some broken parts due to the dryness during SEM sample preparation. Its surface was more even and smoother without any granules suggesting it was much more purified than the EBN solution. SDS-PAGE analysis showed that 2.5% EBN solution only displayed bands at the position between >250 kD to 131 kD suggesting that the size of most of its proteins was large. In contrast, 2.5% EBND has a gradual-sized protein distribution from <10 kD to >250 kD. Four bands showed up at the positions of 66, 39, 34 and 12 kD, respectively. The enzyme-only solution showed two bands at 14 and 18 kD as expected (Figure 1C). This data suggested that large-sized proteins in EBN were well hydrolyzed by enzymes in a 2.5% EBND solution.

### 3.2. ORAC Capacity of EBN, EBND and SA under Cell-Free Conditions

Next, we tested the antioxidant activity of EBN, EBND and SA by using the ORAC assay. Figure 1E showed that EBND exhibited excellent antioxidant activity in a concentration-dependent manner. ORAC values of EBND at concentrations of 50, 125 and 250 µg/mL were equaled to 16.2, 53.5 and 95.2 µM of trolox, respectively. Interestingly, EBN solutions at the same concentrations almost did not have any ORAC value. The enzyme-only solution at the lowest dilution rate presented a low ORAC value equaled 11.7 µM of trolox. This data demonstrated that without enzyme digestion, the antioxidant compounds (such as sulfur-containing amino acids) in EBN were not able to release into the solution. Therefore, we used EBND for the rest of our research. Figure 1F showed that SA also presented antioxidant activity in a concentration-dependent manner. ORAC values of SA at concentrations of 1250, 2500, 5000, 12,500 and 25,000 µg/mL were equaled to 5.44, 7.46, 23.4, 81.0 and 158.3 µM of trolox, respectively.

### 3.3. Effects of EBND and SA on Cell Viability and Cellular ORAC Capacity in HaCaT Cells

Before we test the protective effects of EBND and SA to HaCaT keratinocytes and 3D epithelium equivalents, we examined cytotoxicity of them by PrestoBlue assay. Our results showed that after being cultivated with HaCaT cells for 48 h, SA at 2500 µg/mL significantly decreased cell viability (*p <* 0.05). Concentrations of 1250 µg/mL and below did not negatively affect cell viability in HaCaT cells (Supplemental Figure S1A). EBND at the concentrations of 5–250 µg/mL did not show any significant cytotoxicity to HaCaT cells (Supplemental Figure S1B). Thus, we chose concentrations of 250–1250 µg/mL for SA, and 50–250 µg/mL for EBND for the rest experiments.

Since the cellular antioxidant defense system serves to counterbalance oxidative stress, we checked if EBND and SA enhanced cellular antioxidant ability in HaCaT cells. Table 1 showed that after being incubated with EBND (50 and 125 µg/mL) and SA (1250 µg/mL) for 48 h, cellular ORAC capacity was significantly increased. This data suggested that EBND and SA not only have excellent antioxidant activity but also can improve cellular antioxidant capacity in HaCaT cells.

### 3.4. EBND Protects Air-Drying- and UV- Induced Membrane Damage and Cell Death in HaCaT Cells

**Figure 2:**
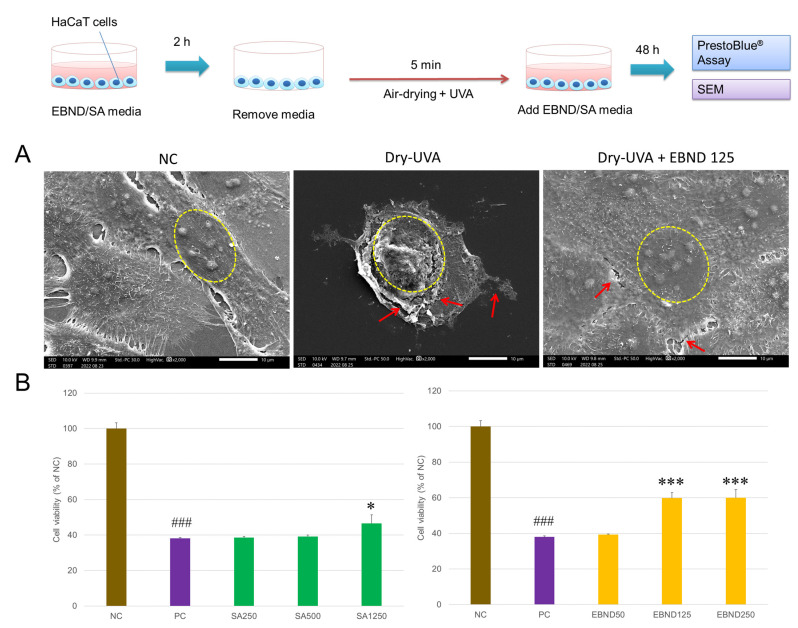
Effects of EBND and SA on air-drying- and UVA- induced cellular membrane damage and cell death in HaCaT cells. The top illustration represents the procedures for the experiments. HaCaT cells (2 × 10^4^ cells/well) were seeded in 35 mm^2^ 4-chamber glass bottom dishes and treated with SA and EBND. At the end of the experiments, the microstructure of cell surfaces was observed by SEM. Cell viability was measured by PrestoBlue assay. (**A**) Typical SEM images of HaCaT cells at 48 h after UVA irradiation. Yellow circles indicate nuclei. Membrane breakages of cells were shown by red arrowheads. Scale bar = 10 µm; (**B**) Cell viability of HaCaT cells. The negative control (NC) cells were cultivated in the maintenance medium without air-drying and UVA irradiation. The positive control (PC) cells were air-dried and UVA-irradiated without EBND or SA treatment. SA250, SA 250 µg/mL; SA500, SA 500 µg/mL; SA1250, SA 1250 µg/mL; EBND 250, EBND 250 µg/mL; EBND 125, EBND 125 µg/mL; EBND 50, EBND 50 µg/mL. The rests are the same. ### *p <* 0.001 vs. NC; * *p <* 0.05, *** *p* < 0.001 vs. PC. Each bar represents the mean ± SD of the three independent experiments.

In our previous study, we demonstrated that both UVA irradiation and air-drying could individually cause severe cellular damage to cells [9,17]. However, cell viability was not significantly decreased when UVA at the dose below 15 J/cm^2^ or the air-drying time was shorter than 10 min. 5 min air-drying only decreased 8% cell viability in HaCaT keratinocytes [9,17]. Here, we combined UV irradiation and air-drying to damage HaCaT cells. The dose of UVA was as low as 10 J/cm^2^ and air-drying time was 5 min. As shown in Supplemental Figure S1A, the phase-contrast microscopic image presented that some bleb-/blister-like protrusions appeared on the surface of HaCaT cells indicating membrane damage (Supplemental Figure S2A, middle panel). SEM analysis showed that compared to the negative control (NC), air-dried and UVA-irradiated HaCaT cells (Dry-UVA) presented cellular membrane breakages (Figure 2A) which were more severe than air-drying damage alone [9]. Although Dry-UVA slightly damaged the intercellular membrane in EBND (125 µg/mL)-treated cells, their cellular membranes were intact (Figure 2A, left panel).

The PrestoBlue^®^ assay showed that Dry-UVA significantly decreased cell viability to 38.1% ± 0.45% of that in NC (Figure 2B). This data indicated that the combination of UVA and air-drying could severely damage cellular membranes and suppress cell proliferation. SA at its highest concentration (1250 µg/mL) significantly increased cell viability to 46.6%, whereas SA at lower concentrations (250 and 500 µg/mL) did not show any protection to HaCaT cells (Figure 2B). Meanwhile, when HaCaT cells were treated with EBND at concentrations of 125 and 250 µg/mL, the cell viability increased to about 60% (*p <* 0.001). Because SA at 1250 µg/mL and EBND at 125 µg/mL showed better protection than other concentrations, we chose these two concentrations for the rest of our Dry-UVA experiments.

**Figure 3:**
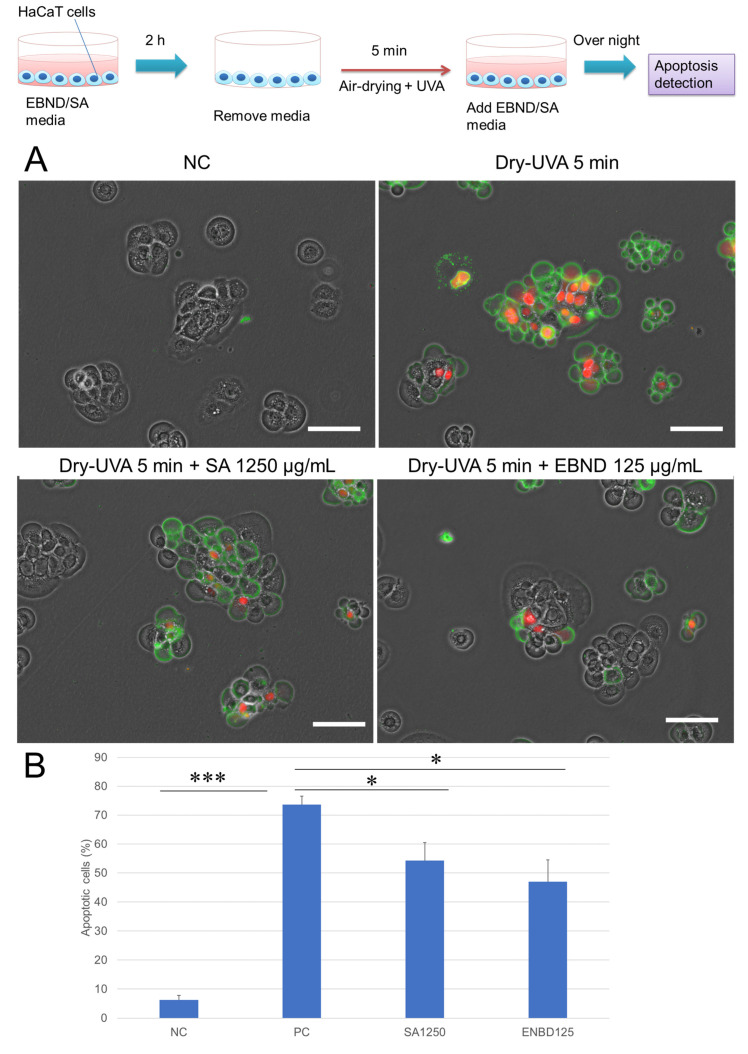
Effects of EBND and SA on Dry-UVA-induced apoptosis in HaCaT cells. The top illustration represents the procedures for the experiments. HaCaT cells were treated in the same manner as in the description in Figure 2. At the end of the experiments, apoptotic cells were detected by Annexin V/PI staining. (**A**) Typical images of Annexin V/PI staining. Green, Annexin V. Red, PI. Scale bar = 50 µm; (**B**) Results of apoptotic cells (analyzed by Tali imaging cytometer). * *p* < 0.05, *** *p <* 0.001 vs. PC. Each bar represents the mean ± SD of the three independent experiments.

We further examined the protective effects of EBND and SA on Dry-UVA-induced apoptosis in HaCaT cells. Figure 3A showed that compared to the negative control, Dry-UVA caused a majority of HaCaT cells to undergo apoptosis. Some nuclei were stained by PI indicating damage to cellular membranes. With SA (1250 µg/mL)- and EBND (125 µg/mL)-treatment, the apoptotic cells were fewer than in the positive control. Analysis from Tali imaging cytometer showed that there were only 6.3% apoptotic cells in the NC cells. Dry-UVA induced 73.7% of cells to suffer apoptosis. Both SA (1250 µg/mL) and EBND (125 µg/mL) significantly reduced the number of apoptotic cells to 54.3% and 47.0%, respectively (Figure 3B).

These data suggested that EBND could protect HaCaT keratinocytes from Dry-UVA-induced cellular membrane damage and cell death.

### 3.5. EBND Diminishes Dryness and UVA-Induced Intracellular ROS and DNA Double-Strand Break in HaCaT Cells

**Figure 4:**
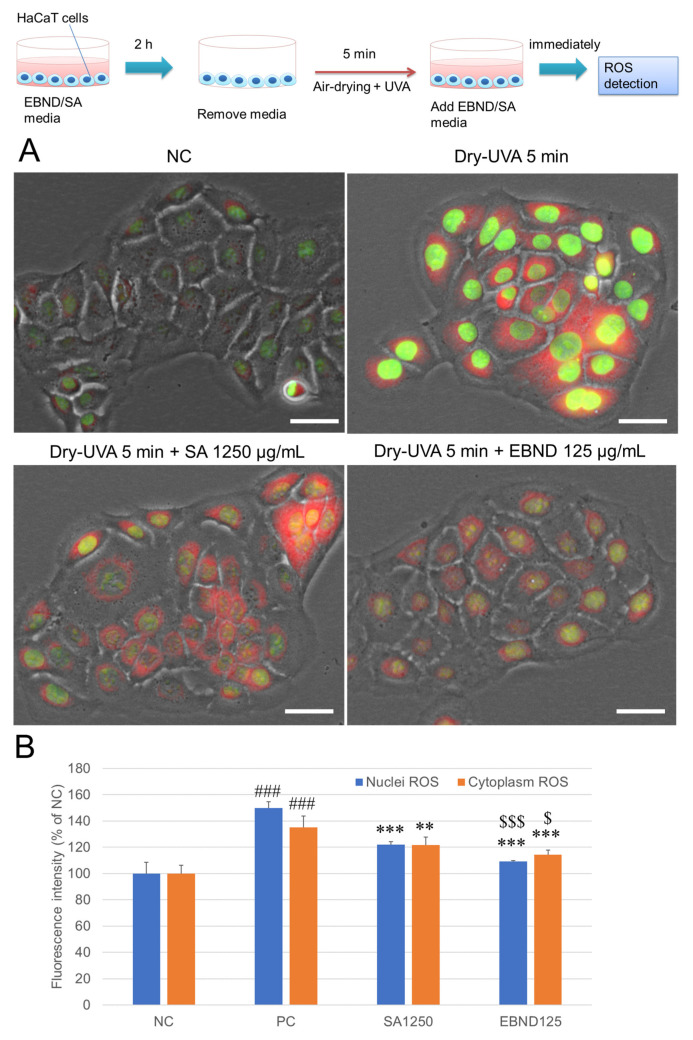
Effects of EBND and SA on air-drying- and UVA-caused intracellular ROS production in HaCaT keratinocytes. The top illustration represents the experimental procedures. (**A**) Typical fluorescence images of HaCaT cells displayed that 5 min air-dried and UVA irradiated HaCaT cells produced intracellular ROS. With EBND or SA treatment, intracellular ROS was significantly suppressed. Green, nuclei ROS; red, cytoplasm ROS. Scale bars = 100 µm; (**B**) Quantitative analysis of cellular ROS. Intensity of green and red fluorescence was analyzed with Tali image-based cytometer as described in “Materials and Methods”. ### *p <* 0.001 vs. NC; ** *p <* 0.01, *** *p* < 0.001 vs. PC; $, *p <* 0.05, $$$, *p <* 0.001, vs. SA1250. Each bar represents the mean ± SD of the three independent experiments.

We then investigated the effect of EBND and SA on dryness and UVA-induced oxidative stress and DNA damage in HaCaT cells. As shown in Figure 4A,B, after Dry-UVA ROS production was significantly increased in both nuclei and cytoplasm of HaCaT cells. When HaCaT cells were pre-treated with EBND (125 µg/mL) or SA (1250 µg/mL) for 2 h, both nuclei and cytoplasm ROS were significantly diminished (*p <* 0.001 and *p <* 0.01). EBND exhibited significantly better effects than SA.

**Figure 5:**
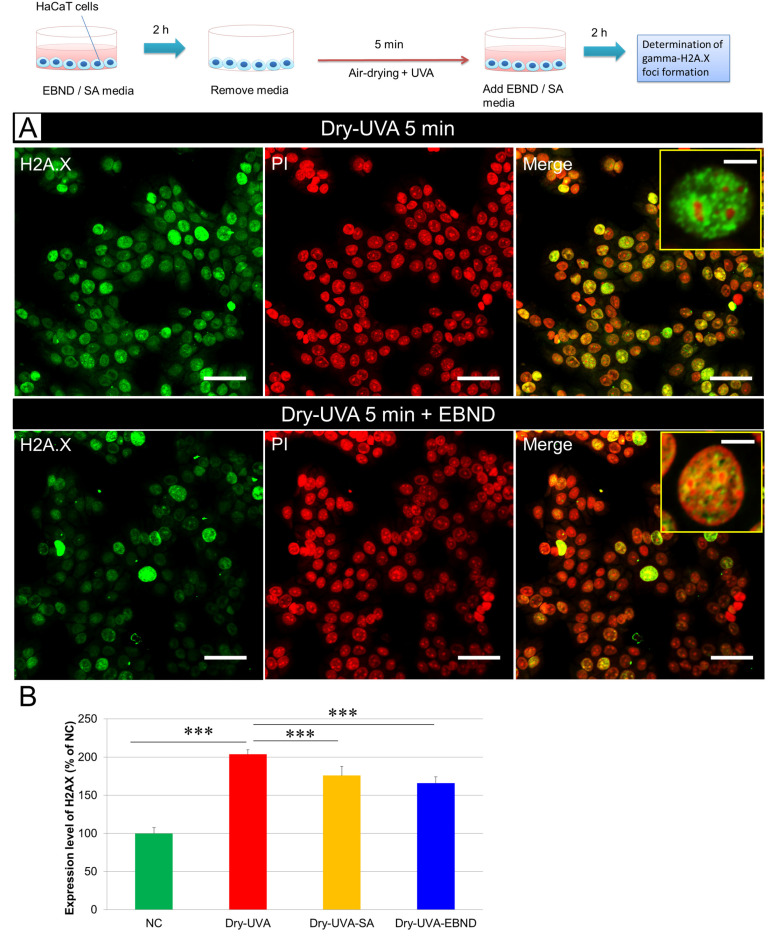
Effect of EBND and SA on Dry-UVA-induced DSBs in HaCaT cells. The top illustration represents the experimental procedures. (**A**) Typical images of anti-H2A.X antibody-based immunofluorescence staining. Green, γ-H2A.X; Red, nuclei (PI). The upper panel shows that 5 min air-drying and UVA irradiation-induced many H2A.X foci formation in HaCaT cells indicating DSBs. The enlarged single-cell image in the upper-right corner displayed many green spots (typical H2A.X foci) in the nucleus. The lower panel shows that with EBND treatment (125 µg/mL), the expression level of H2A.X was significantly lower. There are fewer green spots in the nucleus of a cell in the enlarged image in the right corner. Scale bars indicate 15 µm (bigger images) and 5 µm (smaller images in the upper-right corner; (**B**) Quantitative analysis of the expression level of H2A.X with Tali image-based cytometer as described in “Materials and Methods”. Data represent the mean ± SD of the three independent experiments. *** *p <* 0.001.

One of the severe cellular injuries caused by ROS was DNA double-strand break (DSBs). We further investigated if EBND and SA can reduce Dry-UVA-induced DSBs in HaCaT cells. As shown in Figure 5A,B, at 2r after Dry-UVA, HaCaT cells generated significantly higher amounts of γ-H2A.X foci (the sign of DSBs) than that in the NC cells suggesting severe DNA damage in HaCaT cells (*p <* 0.001). Both SA (1250 µg/mL) and EBND (125 µg/mL) significantly reduced DSBs as seen by the decreased green spots (*p <* 0.01). These data suggested that both EBND and SA could suppress UVA and dryness-induced oxidative stress and therefore protect HaCaT cells from DNA damage caused by ROS.

### 3.6. EBND Diminishes Dryness and UVA-Induced Apoptosis and ROS Production in 3D Epithelium Equivalents

**Figure 6:**
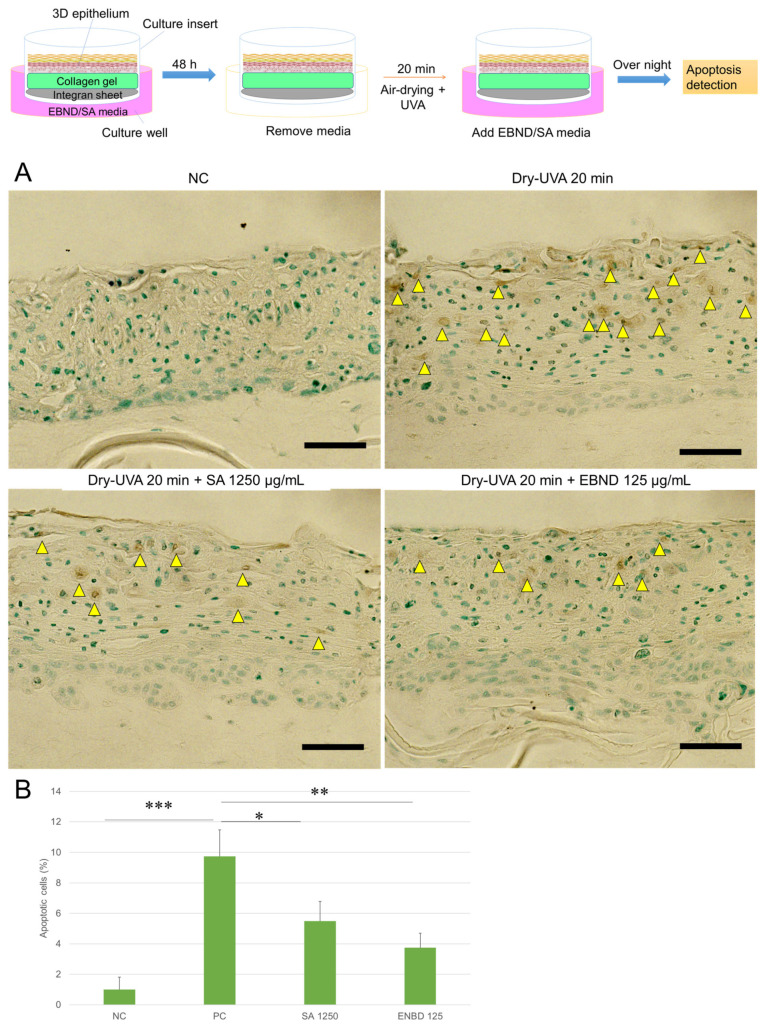
Effects of EBND and SA on Dry-UVA- induced apoptosis in 3D epithelium equivalents. The top illustration represents the experimental procedures. The epitheliums were treated in the same manner as in the description in “Materials and Methods”. At the end of the experiments, apoptotic cells were detected by TUNEL staining. (**A**) Typical images of TUNEL staining. Yellow arrows indicate apoptotic cells. Blue, nuclei. Brown, apoptotic cells. Scale bar = 100 µm; (**B**) Quantitative analysis of apoptotic cells (analyzed by Image J software). * *p* < 0.05, ** *p <* 0.01, *** *p <* 0.001. Each bar represents the mean ± SD of the three independent experiments.

We then investigated the protective effects of EBND and SA against dryness and UVA-caused injuries in reconstructed 3D epithelium equivalents. As shown in Figure 6A and Figure S3, the epitheliums exhibited a stratified squamous epithelial surface with a stratum corneum-like thin layer. There were almost no apoptotic cells in the negative control tissue. After being exposed to dryness and UVA irradiation for 20 min, some cells underwent apoptosis. When the epitheliums were pre-treated with EBND (125 µg/mL) or SA (1250 µg/mL) for 48 h, the number of apoptotic cells decreased. Image J analysis showed that only 1% of the cells in the epithelium underwent apoptosis without Dry-UVA exposure (NC). Dry-UVA caused about 10% of the cells to suffer apoptosis (PC) in the tissue. SA and EBND significantly reduced apoptotic cells to 5.5% and 3.75%, respectively (*p <* 0.05 and *p <* 0.01) (Figure 6B).

**Figure 7:**
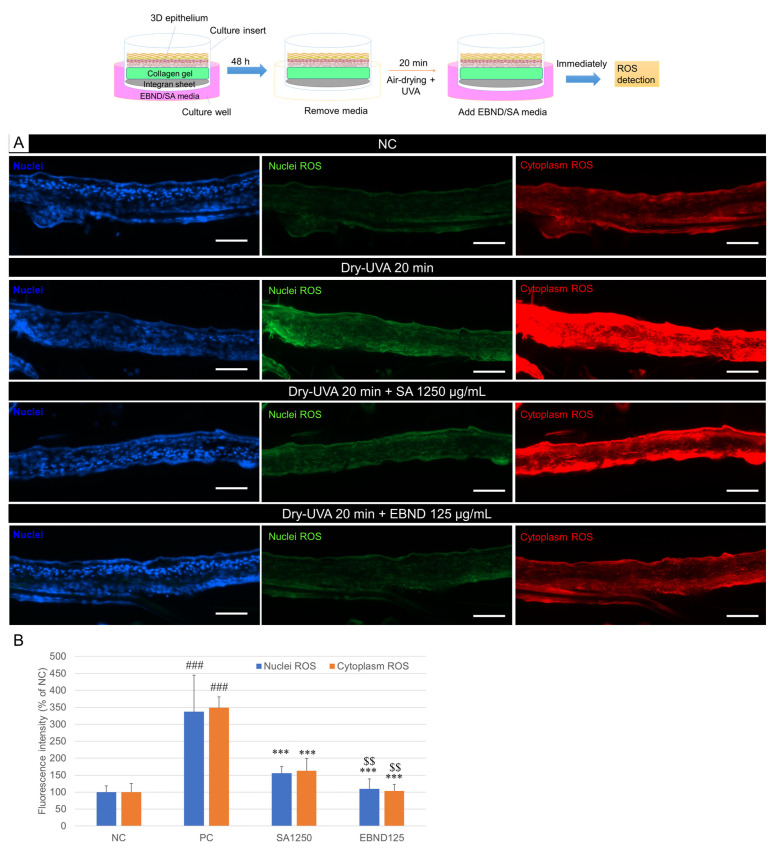
Effects of EBND and SA on Dry-UVA- induced ROS in 3D epithelium equivalents. The top illustration represents the experimental procedures. (**A**) Typical images of CellROX^®^ orange and green staining. Blue: Hoechst 33342; Green: nuclei ROS; Red: cytoplasm ROS. Scale bar = 200 µm (**B**) Quantitative analysis of cellular ROS. Intensity of green and red fluorescence was analyzed with Image J software. ### *p <* 0.001 vs. NC; *** *p* < 0.001 vs. PC; $$, *p <* 0.01, vs. SA1250. Each bar represents the mean ± SD of the three independent experiments.

Next, we checked if EBND and SA suppress Dry-UVA-induced ROS in 3D epithelium equivalents. Figure 7A showed that compared to the negative control (NC), both nuclei and cytoplasm ROS in the epitheliums were observably increased by 20 min Dry-UVA. SA and EBND treatments suppressed ROS production in the tissue, especially EBND treatment. Image J analysis showed that Dry-UVA increased nuclei and cytoplasm ROS production to 337.4% and 349.1% of that in the NC, respectively. Both SA (1250 µg/mL) and EBND (125 µg/mL) significantly reduced nuclei ROS to 156.0% and 109.9%, and cytoplasm ROS to 163.2% and 103.3%, respectively. EBND exhibited significantly better effects than SA (Figure 7B).

These data suggest that both EBND and SA could suppress Dry-UVA-induced apoptosis and ROS generation in 3D epithelium equivalents. EBND showed superior effects to SA.

### 3.7. EBND Suppresses UVB-Induced Inflammatory Cytokines Secretion in HaCaT Cells

**Figure 8:**
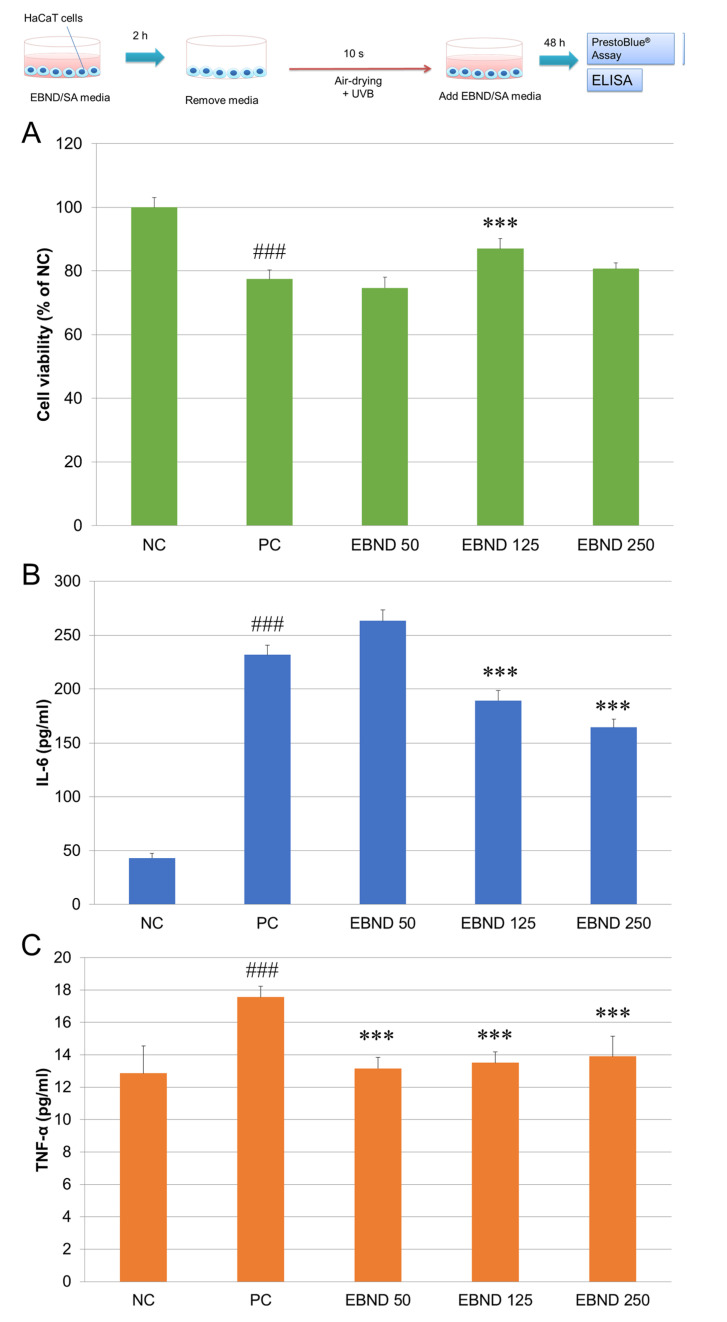
Repressive effects of EBND on UVB-induced cell proliferation suppression and IL-6 and TNF-α secretion in HaCaT cells. The top illustration represents the experimental procedures. At the end of cultivation, cell viability was measured by PrestoBlue assay. Concentrations of IL-6 and TNF-alpha in cell culture supernatant were measured by ELISA as described in Materials and Methods. (**A**) Cell viability of HaCaT cells; (**B**) Human IL-6 level in the culture supernatants of HaCaT cells was determined by ELISA; (**C**) Human TNF-α level determined by ELISA. The negative control (NC) cells were cultivated in the maintenance medium without air-drying and UVB irradiation. The positive control (PC) cells were air-dried and UVB-irradiated without EBND treatment. ### *p <* 0.001 vs. NC. *** *p* < 0.001 vs. PC. Each bar represents the mean ± SD of the three independent experiments.

UVB is known to induce inflammatory responses in the skin. Therefore, we tested if EBND could suppress UVB-induced inflammatory responses in HaCaT cells. We first tested the protective effect of EBND on UVB-induced cell death in HaCaT cells. During UVB irradiation, the medium was sucked up. So, the cells were also air-dried. However, because the time period was only 10 s, air-drying did not cause obvious membrane damage like the 5 min air-drying did (Supplemental Figure S2B, middle panel vs. A, middle panel). The effects of air-drying can be ignored. UVB irradiation (16 mJ/cm^2^) significantly suppressed cell viability to 77.5% of that in the NC cells. Treatment with EBND at concentrations of 125 and 250 µg/mL significantly increased the cell viability to 87.0% and 80.7% (*p <* 0.001), respectively (Figure 8A). These data suggested that EBND could protect UVB-induced cell death in HaCaT cells, especially at the concentration of 125 µg/mL.

Next, we measured the suppressive effects of EBND in UVB-induced inflammatory responses. As shown in Figure 8A,B, UVB irradiation significantly increased the secretion of both IL-6 and TNF-α, especially since the amount of IL-6 was increased 5 times more than the NC. EBND at concentrations of 125 and 250 µg/mL significantly decreased the secretion of IL-6 (*p <* 0.001) whereas EBND at 50 µg/mL slightly increased the amount of IL-6. The amount of TNF-α was also significantly decreased by EBND at all three concentrations of 50, 125 and 250 µg/mL (*p <* 0.001). These data suggested that EBND could suppress UVB-induced inflammatory responses in HaCaT keratinocytes.

## 4. Discussion

EBN contains proteins, sialic acid (SA), polyunsaturated fatty acids (PUFA), minerals and other compounds [23]. Proteomic analysis showed that 398 proteins have been identified in EBN [24]. Previous studies reported that EBN exhibited antiviral, immune-regulative, neuroprotective, antioxidant, anti-inflammatory and anti-aging effects [25]. The main bioactive compounds in EBN are SA, PUFA and proteins-derived amino acids [26,27,28]. Most of these bioactive compounds bind to or form large-sized molecules. Without hydrolysis, they cannot be activated. In this present study, by using SEM and SDS-PAGE analysis, we demonstrated that after enzyme digestion, the large-sized molecules can be hydrolyzed into much smaller molecules. Similar to a previous report [29], the digested EBND solution exhibited excellent ORAC capacity whereas the non-digested EBN solution did not, suggesting that bioactive compounds were released by enzyme hydrolyzation. Since SA is known as one of the main bioactive compounds in EBN, we use SA as the control reagent. ORAC assay showed that SA has antioxidant activity. However, compared to EBND, its ORAC capacity was much lower. The ORAC value of EBND at 125 µg/mL was equivalent to 53.5 µM trolox whereas SA at 1250 µg/mL was only equivable to 5.44 µM trolox. This result also indicated that the antioxidant activity in EBND is not from SA, but rather from other bioactive compounds.

As the main contributor to skin ageing, UV triggers oxidative stress which causes lesions to cellular compounds (including lipids, proteins and DNA). UV is also known to accelerate transepidermal water loss in the skin [30,31], especially in arid climates. However, little is known about the effects of the combination of UV and an arid environment on cells. In this present study, our data first demonstrated that the combination of non-toxic UVA irradiation and 5 min air-drying could bring severe membrane damage, cell death, ROS generation and DSBs in HaCaT keratinocytes. These data suggested that UVA can be more toxic in arid climates. At the tissue level, Dry-UVA could significantly induce apoptotic cell death and ROS generation in 3D epithelium equivalents. Nevertheless, the administration of EBND could significantly suppress the above-mentioned lesions in both HaCaT cells and the epitheliums, especially at the concentration of 125 µg/mL.

Cellular antioxidant defense systems contain enzymatic and nonenzymatic antioxidants, such as superoxide dismutase, catalase, vitamins and glutathione. These cellular antioxidants scavenge ROS and protect cells/tissues from oxidative stress [32]. Our data demonstrated that EBND could significantly increase cellular antioxidant capacity. Its effect was similar to SA at a 10 times lower concentration. This result suggested that EBND achieves its cytoprotective effects partially through the enhancement of cellular antioxidant defense systems.

UVB is the main reason for erythema (an inflammatory response in the skin). Since keratinocytes-formed stratum corneum absorbs almost all UVB, keratinocytes are the first target of UVB radiation. Strong and chronic UVB exposure initiates pro-inflammatory cytokines secretion and damages the barrier function of keratinocytes [33]. Our results from ELISA and cell viability assay showed that UVB (16 mJ/cm^2^) could markedly increase the secretion of pro-inflammatory cytokines (IL-6 and TNF-α) and inhibit cell proliferation in HaCaT keratinocytes suggesting that UVB initiated inflammatory responses and induced cell death. EBND administration significantly suppressed UVB-induced cell death and IL-6 and TNF-α secretion suggesting that EBND can protect keratinocytes from UVB-induced harmful effects.

SA, the best-known bioactive compound of EBN, is typically found at the outermost end of glycan chains on cellular surfaces. SA plays important roles in molecular and cellular communication, pathogen recognition and development [34,35]. SA regulates cellular water balance by modulating voltage-gated sodium and potassium channels [36,37]. SA also exhibited anti-inflammatory properties [38,39]. Thus, the protective effects of EBND against dryness- and UV-induced injuries are supposed to be from SA. However, our data demonstrated that exogenous SA treatment to HaCaT cells and 3D epithelium equivalents did not show better effects than EBDN even at 10 times higher concentrations. Since EBND contains about 10% SA, the concentration of SA in EBND were 100 times lower than the exogenous SA. Thus, the protective effects of EBND are probably not from SA but from other bioactive compounds.

It has been reported that EBN extracts contain 18 types of amino acids [40]. Some of these amino acids, such as tryptophan, methionine, histidine, lysine, cysteine, arginine and tyrosine, show strong antioxidant activity [41]. Another bioactive compound of EBN, PUFA, also exhibited excellent antioxidant capacity [42]. Thus, the antioxidant compounds in EBND are possibly the above-mentioned amino acids and PUFA. These antioxidant compounds scavenge dryness- and UV-induced ROS, therefore protecting cells and epitheliums from oxidative stress-caused DNA damage and cell death. Additionally, PUFA and some EBN-derived amino acids also have anti-inflammatory properties [43,44]. They probably reduced UVB-induced inflammatory responses in HaCaT cells.

In conclusion, our data demonstrated that enzyme digestion could release the bioactive compounds (including SA, amino acids and PUFA) in EBN. EBND exhibited antioxidative properties; increased cellular antioxidant capacity; protected HaCaT keratinocytes and 3D epithelium equivalents from dryness- and UV- induced DSBs, membrane damage and cell death; and reduced inflammatory responses. Its effects were significantly greater than SA. EBND can be applied as a skin-care reagent.

## Figures and Tables

**Table 1:** Effects of EBND and SA on cellular ORAC capacity in HaCaT cells.

Experimental Group (n = 8)	ORAC Value [Trolox Equivalents (µM) (Mean ± SD)]	*p* Value (vs. NC)
NC	166.7 ± 3.51	
SA 250 µg/mL	169.9 ± 3.03	1.000
SA 500 µg/mL	172.4 ± 2.65	0.055
SA 1250 µg/mL	176.1 ± 2.87	0.001 **
EBND 50 µg/mL	177.5 ± 1.69	0.000 ***
EBND 125 µg/mL	176.7 ± 2.22	0.000 ***
EBND 250 µg/mL	172.8 ± 3.56	0.088

HaCaT cells (1 × 10^6^ cells) were seeded into 60 mm^2^ culture dishes and incubated with SA and EBND for 48 h. At the end of incubation, cells were lysed and the extracts were followed by ORAC assay the same as in the description in “Materials and Methods”. NC, the negative control; ** *p <* 0.01, *** *p <* 0.001 vs. NC.

## Data Availability

The data presented in this study are available within the article and its Supplementary Materials. Other data related to this study are available on request from the corresponding author.

## Supplementary Materials

The following supporting information can be downloaded at: https://www.mdpi.com/article/10.3390/antiox12030609/s1, Figure S1. Effects of EBND and SA on cell viability in HaCaT cells; Figure S2. Morphological changes of HaCaT cells after Dry-UVA- and UVB-exposure; Figure S3. H&E staining for 3D epithelium equivalents.
