# Supplementary material for: Enzyme-Digested Edible Bird’s Nest (EBND) Prevents UV and Arid Environment-Induced Cellular Oxidative Stress, Cell Death and DNA Damage in Human Skin Keratinocytes and Three-Dimensional Epithelium Equivalents
Source: Antioxidants (Basel). 2023 Mar 1;12(3):609. doi: 10.3390/antiox12030609 (PMC10045731; doi:10.3390/antiox12030609)
Supplement: Supplementary file 1 [file antioxidants-12-00609-s001.zip › antioxidants-2250224-supplementary.pdf]

Enzyme-digested edible bird's nest (EBND) prevents UV and arid environment-induced cellular oxidative stress, cell death and DNA damage in human skin keratinocytes and three-dimensional epithelium equivalents

Supplemental materials

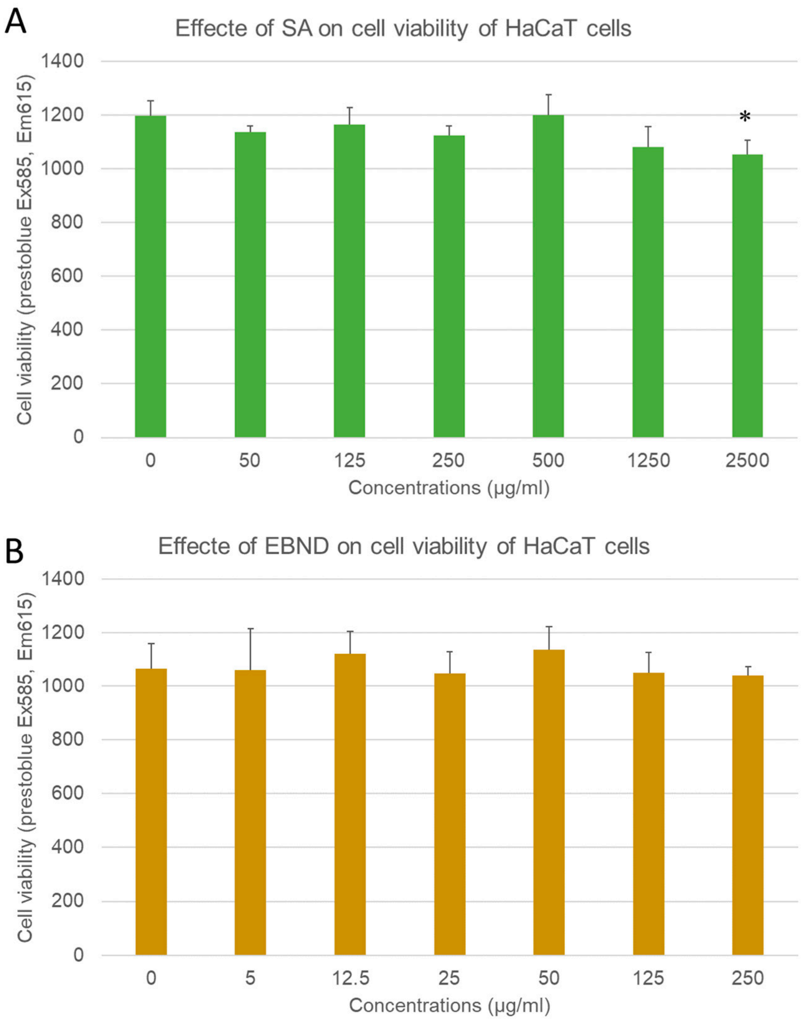

Supplemental Figure 1. Effects of EBND and SA on cell viability in HaCaT cells

HaCaT cells (1×10^4 cells/well) were seeded into 24-well plates and then incubated with SA and EBND at different concentrations for 48 hr. At the end of cultivation, cell viability was measured by ProstoBlue assy. *, $p<0.05$ v.s. 0. Each bar represents the mean ± SD of three independent experiments.

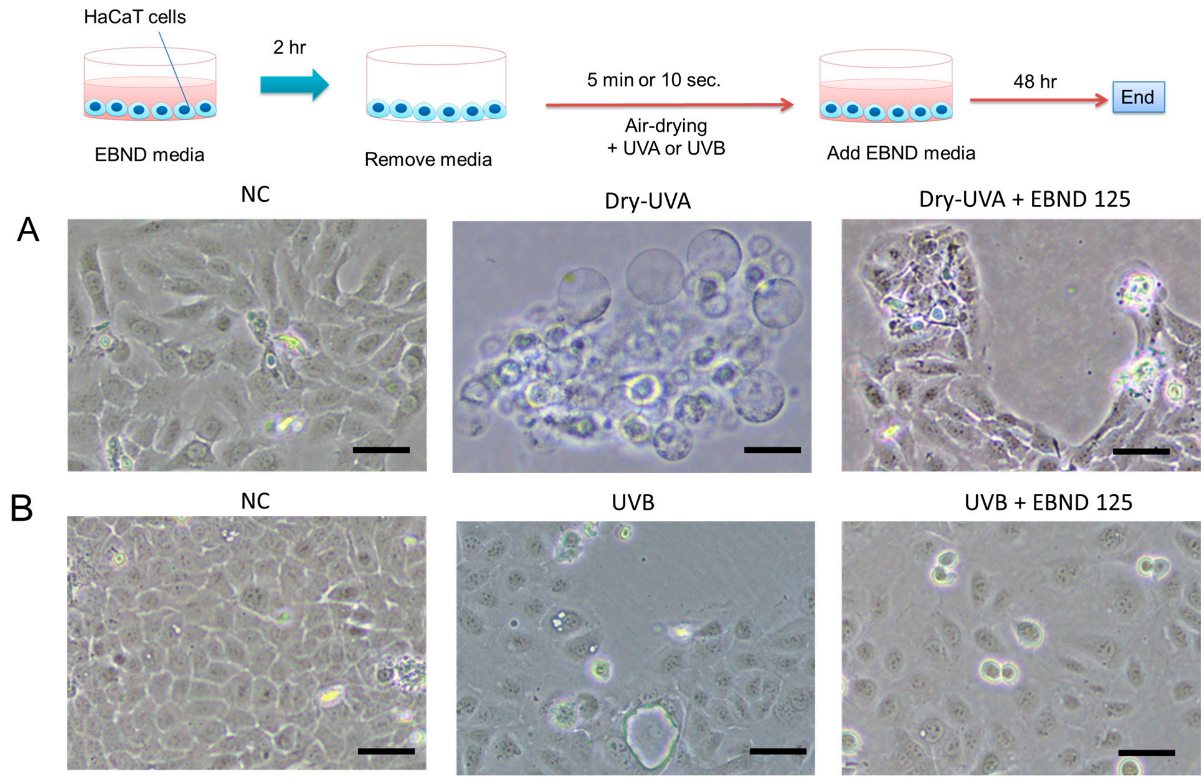

Supplemental Figure 2. Morphological changes of HaCaT cells after Dry-UVA- and UVB-exposure

Illustration on the top represents procedures of the experiments. HaCaT cells (4×10^4/well) were seeded into 24-well plates and incubated with EBND (125 µg/ml) for 2hr. Cells were then undergoing Dry-UVA- or UVB-exposure the same as the description in "Materials and Methods". (A) Typical phase-contrast microscopic images of HaCaT cells 48 hr after Dry-UVA-exposure. (B) Typical phase-contrast microscopic images of HaCaT cells 48 hr after UVB irradiation. All scale bars = 50 µm.

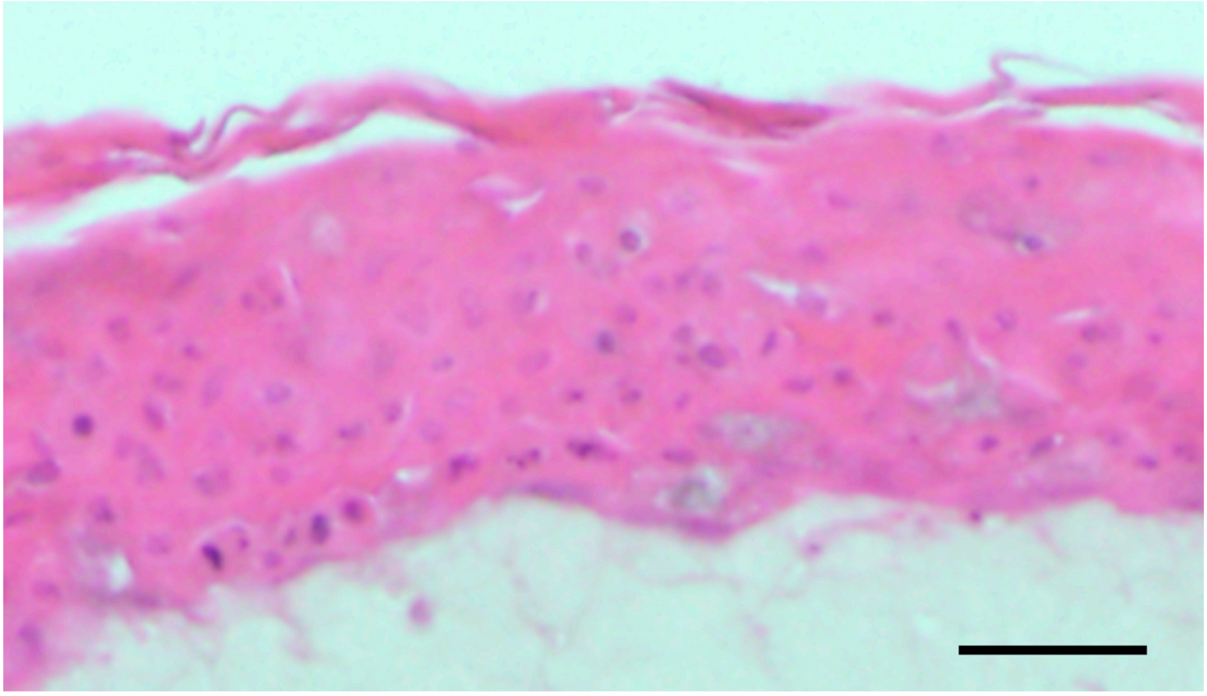

Supplemental Figure 3. H&E staining for 3D epithelium equivalents

The 3D epithelium equivalents were reconstructed the same as the description in "Materials and Methods". At the end of cultivation, the tissues were fixed by 10% Formalin Neutral Buffer Solution at 4 °C overnight. The samples were then dehydrated by a series of ethanol and xylene and followed by paraffin embedding. The paraffin tissue blocks were sectioned to 10 µm-thick slices by a microtome. After deparaffinization and hydration, the samples were stained by H&E reagents. Typical image shows the 3D epithelium without any treatment. Scale bars = 100 µm.
